# Electrostatic Effects on Tau Nanocondensates

**DOI:** 10.3390/biom15030406

**Published:** 2025-03-12

**Authors:** Phoebe S. Tsoi, Lathan Lucas, Derek Rhoades, Josephine C. Ferreon, Allan Chris M. Ferreon

**Affiliations:** 1 Department of Biochemistry and Molecular Pharmacology, Baylor College of Medicine, Houston, TX 77030, USA; phoebe.tsoi@bcm.edu (P.S.T.); lathan.lucas@bcm.edu (L.L.); 2 Department of Chemistry, Scripps Research, La Jolla, CA 92037, USA; drhoades@scripps.edu

**Keywords:** Tau, biomolecular condensates, LLPS, nanocondensates, neurodegeneration, protein condensation, tauopathy

## Abstract

Biomolecular condensates (BMCs) are membrane-less protein compartments with physiological and pathological relevance. The formation of BMCs is driven by a process known as liquid–liquid phase separation (LLPS), a field that has largely focused on the study of micron-sized condensates. However, there have been recent studies showing that proteins that undergo LLPS also form nanometer-sized condensates. These nanometer-sized condensates, or nanocondensates, are distinct from microcondensates and potentially exhibit more relevance in cell biology. The field of nanocondensate research is in its infancy, with limited biophysical studies of these structures. Here, we studied condensate formation and dissolution of wild-type and disease-linked (hyperphosphorylated and missense mutated) Tau. We investigated the effects of solution condition modulation on nanocondensate formation and dissolution, and observed that Tau condensation is strongly regulated by electrostatic forces and less affected by hydrophobic disruption. We observed that all three Tau variants studied shared condensate formation properties when in solution conditions with the same ionic strength. However, hyperphosphorylated and missense-mutated Tau exhibited higher resistance to dissolution compared to wild-type Tau. This study uncovers additional distinctions between different types of condensates, which provides further insight into the distinctions between physiological and pathological condensates.

## 1. Introduction

Tau is a protein intricately involved in neuronal health; its physiological relevance in neuronal microtubule stabilization [1,2,3,4,5,6,7] is juxtaposed by its implication in neurodegeneration [3,7,8,9,10,11]. In both physiological and pathological contexts, condensation plays a significant role. Tau condensates regulate microtubule assembly [2,3,4,6], but have also been shown to seed the formation of toxic oligomers that develop into amyloidal aggregates implicated in neurodegenerative disease [3,9,11,12].

Tau condensation has been a significant field of research in recent years, with the bulk of research focusing on microcondensates (micron-sized condensates). Condensation is driven by liquid–liquid phase separation (LLPS), a process studied in vitro using optical microscopy and turbidity assays [13,14] and thought to be initiated by classical nucleation theory. Classical nucleation postulates that molecules in solution above a calculatable saturated concentration will favor the formation of a new phase [15,16,17] and the probability of molecular assemblies at subsaturated concentrations is negligibly small [18]. Recently, however, nanometer-sized condensates have been observed in subsaturated concentrations for many proteins, including lysozyme [19,20], Tau [2], and FUS [18]. Apart from size, there has been minimal research investigating the distinction between nanometer-sized and micrometer-sized condensates.

Tau nanocondensates (nanometer-sized condensates) have been shown to have physiological roles in synaptic vesicle regulation [4]. Tau condensates that form on microtubules are also nanometer in size [2,5]. Tau nanocondensates are a relatively new field of research, with limited efforts made to characterize the forces that regulate the formation and dissolution of these structures. The characterization of nanocondensates could provide more insights into what distinguishes physiological from pathological condensates.

Here, we investigated the formation of nanocondensates using three variants of Tau: wild-type (WT), hyperphosphorylated (pTau), and missense familial mutation (P301S). Tau protein extracted from Alzheimer’s disease patient samples is largely hyperphosphorylated [10,11,21,22,23,24,25], whereas P301S is a familial mutation that leads to degenerative Tauopathy [8,10,11,23,24,26,27,28]. We studied the formation and dissolution of nanocondensates primarily using dynamic light scattering (DLS). All three variants of Tau exhibited hypersensitivity to condensate formation disruption by electrostatics, even at relatively low salt concentrations. The dissolution of condensates, however, varied drastically for each Tau form. WT dissolution occurred at low electrostatic intervention, while pTau required 1000-fold higher salt concentration, and P301S required 100,000-fold higher salt concentration. These results suggest that pTau and P301S nanocondensates resist dissolution, which may indicate a more pathologic characteristic. Interestingly, P301S Tau forms a multimodal intermediate during dissolution, further implying a pathological trait that may explain the distinct pathology linked to familial Tau mutations.

## 2. Materials and Methods

### 2.1. WT, pTau, and P301S Tau Expression and Purification

The bacterial expression and purification of all three forms of Tau were performed as previously described [29]. Briefly, plasmids encoding wild-type Tau, wild-type Tau and GSK3 kinase, and Tau with missense mutation P301S were overexpressed in *E. coli* BL21 star cells and grown at 37 °C in Terrific Broth medium containing carbenicillin (Gold Biotechnology, St. Louis, MO, USA). The culture was induced with 1 mM isopropyl β-d-1-thiogalactopyranoside (IPTG) and grown overnight at 18 °C before harvest via centrifugation at 4000× *g*. Pelleted cells were resuspended in 50 mM NaCl, 5 mM DTT, 50 mM sodium phosphate, pH 6.5, and protease inhibitor cocktail (Xpert Protease Inhibitor, GenDEPOT, Katy, TX, USA), and lysed using a homogenizer (Avestin, Ottawa, ON, Canada). The lysate was centrifuged for 30 min at 50,000× *g* before more salt was added to a final concentration of 450 mM NaCl, and incubated for 30 min in near-boiling water (~85–95 °C). The solution was centrifuged for 2 h at 50,000× *g* and the supernatant was concentrated and then diluted to a final salt concentration of 50 mM NaCl before application to a heparin sepharose HP column (GE, Chicago, IL, USA) for FPLC (Biorad, Hercules, CA, USA) purification, eluted using a salt gradient of 0–600 mM NaCl. Fractions containing Tau protein were collected, concentrated, and further purified using reverse-phase HPLC (C3 column, Agilent, Santa Clara, CA, USA). The purified protein was collected, aliquoted, lyophilized, and stored at −80 °C until later use.

The fully purified protein was run on an SDS-PAGE gel and stained with Coomassie Blue before destaining and subsequent imaging (Supplementary Figure S1).

### 2.2. pTau Verification via Mass Spectroscopy

PTau was boiled in 30 µL of 1× NuPAGE LDS sample buffer (Invitrogen, Carlsbad, CA, USA) and run on SDS-PAGE (NuPAGE 10% Bis-Tris gel, Invitrogen, Waltham, MA, USA), which was visualized with Coomassie Brilliant blue-stain. The SDS-PAGE gel containing the band corresponding to pTau was isolated, destained, and cut using 100 ng trypsin (#T9600, GenDEPOT). The resulting peptide fragments were resuspended in 10 µL of 0.1% formic acid and loaded onto a nanoHPLC-MS/MS system with an EASY-nLC 1200 coupled to a Fusion Tribrid Orbitrap Lumos mass spectrometer (Thermo Fisher, Waltham, MA, USA). The peptides passed through a Reprosil-Pur Basic C18 pre-column (1.9 µm, Dr. Maisch GmbH, Ammerbuch-Entringen, Germany; 2 cm × 100 µm). The pre-column was switched in-line with a 50 mm × 150 µm analytical column, packed in-house with Reprosil-Pur Basic C18 resin, equilibrated in 0.1% formic acid. Peptide fragments were eluted using a discontinuous gradient of 4–28% acetonitrile/0.1% formic acid at a flow rate of 750 nL/min for 45 min. Eluted fragments were electro-sprayed into a mass spectrometer, operated via data-dependent acquisition mode, acquiring fragmentation spectra under the direct control of Xcalibur software (4.0; Thermo Fisher). The parent MS spectrum was acquired with a full MS range of 300–1400 m/z with a resolution of 120,000. The CID fragmented MS/MS spectrum was acquired in ion-trap with rapid scan mode. The obtained MS/MS spectra were matched to the target-decoy human refseq database (June 2015 release, containing 73,637 entries) in Proteome Discoverer 1.4 interface (Thermo Fisher) using the Mascot algorithm (Mascot 2.4, Matrix Science, London, UK). Serine and threonine phosphorylation were allowed. Precursor mass tolerance was confined within 20 ppm with a fragment mass tolerance of 0.5 Da and with a maximum of two missed cleavages allowed. The peptides identified in the Mascot results file were validated with a 5% false discover rate (FDR) and subjected to manual verification to confirm phosphorylation.

### 2.3. pTau Charge Distribution Determination

pTau charge distribution was determined using Prot pi (https://www.protpi.ch; accessed on 23 December 2024). Amino acid sequences corresponding to individual domains were input to the software. Phosphorylation sites at exact positions were denoted and the resulting pI was reported.

### 2.4. Protein Sample Preparation and Concentration Determination

Lyophilized Tau protein was dissolved in water and centrifuged at 20,000× *g* for 10 min before filtration through a 0.02 µm filter. Protein concentrations were determined using the UV absorbance extinction coefficient at 280 nm, calculated using the Edelhoch method [30,31].

### 2.5. Dynamic Light Scattering

Dynamic light scattering (DLS) was performed on the DynaPro Nanostar II (Wyatt Technology, Waters Corporation; Santa Barbara, CA, USA), which measures the Brownian motion of molecules in solution by analyzing intensity fluctuations of scattered light from a 658 nm incident laser. Fluctuations of scattered light are collected by orthogonal detectors and correlated to diffusion coefficient (D), which can be used to determine the hydrodynamic radius (R_h_) through the Stokes–Einstein equation:

$$R_{h}=\frac{kT}{6\pi \eta D}$$

where k is the Boltzmann constant, T is the absolute temperature, and η is the viscosity of the solution.

Samples of varying protein (0.5–4, 20 µM) and salt (0–1 M NaCl) concentrations were prepared in 20 mM HEPES buffer, pH 8, and pipetted into a 45 µL quartz cuvette (Wyatt Technology, Waters Corporation; Santa Barbara, CA, USA). All solutions involved in sample preparation were double-filtered with 0.02 μm filters. Ten DLS acquisitions were taken at 2 s intervals over 10 min at 25 °C for all measurements.

### 2.6. Tau Microcondensate Induction and Microscopy Imaging

In total, 20 μM Tau protein was added to 20 mM HEPES buffer, pH 8, and subsequently imaged via optical microscopy. In total, 5 μL volume was pipetted onto 35 mm glass-bottom dishes (ibidi, Martinsried, Germany) and droplet formation was immediately imaged using an FL EVOS imaging system (Invitrogen).

### 2.7. Electrostatic Effect on Tau Nanocondensate Formation and Dissolution

To assess the effects of electrostatic force on nanocondensate formation, salt (0–1 M NaCl) was added to 20 mM HEPES buffer, pH 8, before its subsequent addition to Tau protein (0.5–4 μM). The mixture was immediately measured by DLS.

To assess the effects of electrostatic force on nanocondensate dissolution, nanocondensates were formed by mixing Tau protein with 20 mM HEPES buffer, pH 8, before subsequent salt (0–1 M) addition. The mixture was immediately measured by DLS.

## 3. Results

### 3.1. Wild-Type, Hyperphosphorylated, and P301S-Mutated Tau Can Exist as Monomers, Nanocondensates, and Microcondensates

**Figure 1:**
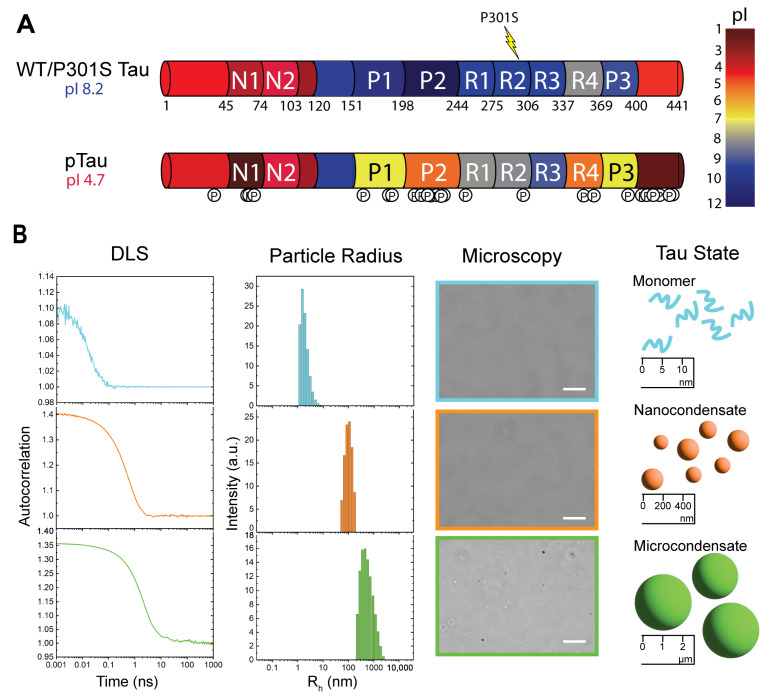
Phase separation of Tau monitored by dynamic light scattering (DLS). (**A**) Domain organizations and charge distributions of wild-type and P301S Tau compared to hyperphosphorylated Tau (pTau), highlighting differences in isoelectric points (pH 8.2 vs. 4.7), and displaying mutation and post-translational modification sites. Tau consists of two N-terminal inserts (N1, N1), three proline-rich domains (P1–P3), and four microtubule-binding repeats (R1–R4). (**B**) Tau can exist as monomers, nanocondensates, and microcondensates, detected using DLS and optical microscopy. Representative data from wild-type Tau are shown. Solution conditions: 20 μM Tau in water (monomers, 2–10 nm); 1μM Tau in 20 mM HEPES buffer, pH 8 (nanocondensates, 80–300 nm); and 20 μM Tau in 20 mM HEPES buffer, pH 8 (microcondensates, > 700 nm). Scale bars represent 10 μm.

Full-length human Tau (2N4R) consists of 441 amino acids, comprising two N-terminal inserts, three proline-rich domains, and four microtubule binding repeats [1,10,11] (Figure 1A). WT Tau has an isoelectric point (pI) at pH 8.2 and exhibits a distinct charge distribution, with acidic N- and C-terminal regions flanking basic proline-rich and microtubule-binding domains (Figure 1A). P301S Tau shares the same charge distribution with wild-type, with one missense mutation at residue 301. pTau, however, greatly disturbs the charge distribution, lowering overall pI to 4.7. Interestingly, the phosphorylation regions are largely located in the C-terminus and the proline-rich domains.

The phosphorylation of these regions lowers the pI, drastically altering the charge distribution of the protein. Exact phosphorylation sites were verified via mass spectroscopy (Supplementary Figure S2).

WT, pTau, and P301S can exist as monomers, nanocondensates, and microcondensates (i.e., nanometer-sized and micrometer-sized condensates, respectively; Figure 1B). These states can be observed via DLS and exhibit autocorrelation decays that corresponde to distinct ranges of particle radii. Monomers were observed at 20 μM Tau in water, characterized by a sharper decay observed in the autocorrelation plot, which corresponds to a hydrodynamic radius of 2–10 nm. Nanocondensates were observed from 0.5 to 4 μM WT and pTau in 20 mM HEPES buffer, pH 8, and 1–4 μM P301S. Nanocondensates exhibit a slower autocorrelation decay that corresponds to a hydrodynamic radius of 80–300 nm. Microcondensates are detectable via optical microscopy (Figure 1B) and were observed at 20 μM Tau in 20 mM HEPES buffer, pH 8. Microcondensates have the slowest autocorrelation decay of the three states, with an irregular tail that does not reach baseline within the experimental timescale; this autocorrelation decay corresponds to a hydrodynamic radius > 700 nm.

### 3.2. WT Tau Condensates Are Strongly Regulated by Electrostatic Forces

**Figure 2:**
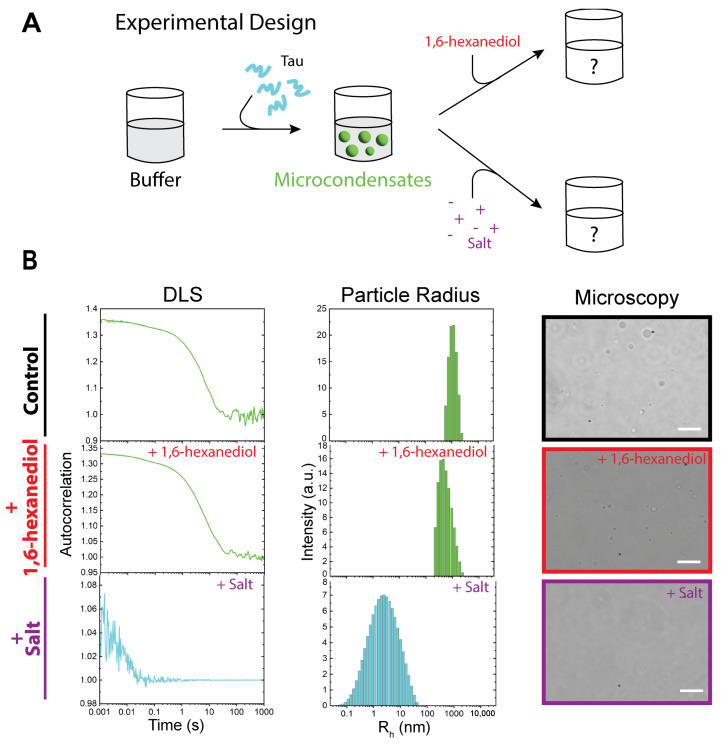
WT Tau microcondensates are modulated by salt, but unperturbed by hexanediol. (**A**) Schematic of salt and hexanediol modulation of Tau microcondensates. (**B**) A total of 20 μM WT Tau in 20 mM HEPES, pH 8, forms microcondensates detected by DLS and optical microscopy. The addition of 5% hexanediol does not significantly affect Tau condensation while the addition of 200 mM NaCl reverses condensates into the monomeric state. Scale bars represent 10 μm.

Biomolecular condensates (BMCs) are characterized by their dynamic and environment-sensitive nature [12,32,33,34,35,36,37,38]. By altering the solution context, we controlled condensate formation and dissolution, and determined the driving forces of Tau liquid enrichment. Previously, we studied hnRNPA1^LCD^ micro- [39] and nanocondensates [40], and determined that micro- and nanocondensates were distinct structures, stabilized by different balances of forces. hnRNPA1^LCD^ microcondensates were readily dissolved using 1,6-hexanediol, and nanocondensates increased in size in the presence of salt [40]. Here, we similarly tested Tau using the same agents (Figure 2A). WT Tau microcondensates were formed by mixing 20 μM protein with 20 mM HEPES buffer, pH 8, and detected via optical microscopy.

Similarly to previous publications [41,42], the addition of 1–10% hexanediol to Tau microcondensates did not result in significant effect (Figure 2B). Microcondensates were still visible using microscopy, and DLS measurements showed very little change in hydrodynamic radius (<30 nm).

Tau condensates, however, appeared to be strongly affected by electrostatic modulation. Incubating the microcondensates in 200 mM NaCl resulted in the dissolution and apparent reversion of the protein to the monomeric state, surprisingly bypassing the nanocondensate state. These results imply a dominant role of electrostatics in the formation and dissolution of nanocondensates.

### 3.3. WT and pTau Share Similar Nanocondensate Formation and Dissolution Properties

**Figure 3:**
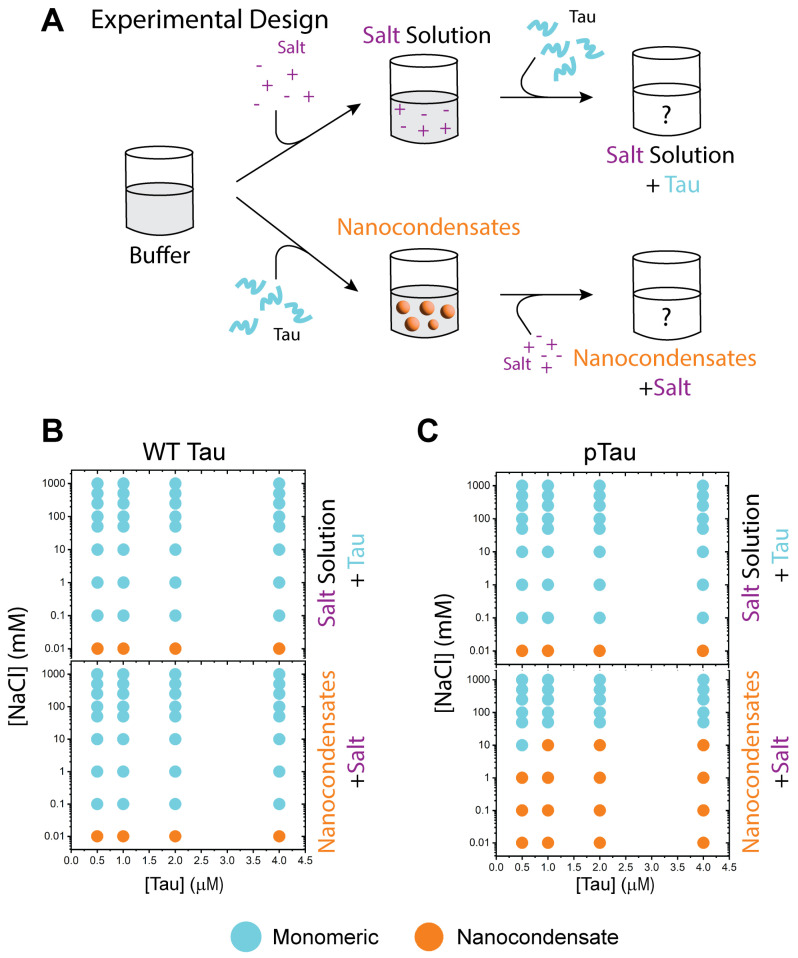
Formation and dissolution of WT Tau and pTau nanocondensates, modulated by salt. (**A**) Schematic of formation and dissolution of Tau condensates via salt modulation. (**B**) Diagram of WT Tau condensate formation (top) and dissolution (bottom) in the presence of salt. (**C**) Diagram of pTau condensate formation (top) and dissolution (bottom) in the presence of salt.

To assess the effects of electrostatic forces on the formation and dissolution of nanocondensates, two sets of experiments were conducted on each Tau variant. Formation was assessed by mixing the buffer (20 mM HEPES, pH 8) with salt (0–1 M NaCl) before its addition to Tau protein. Dissolution was assessed by first forming the nanocondensate (mixing 20 mM HEPES buffer, pH 8, with protein) and then adding in salt (0–1 M) (Figure 3A).

WT Tau condensation and nanocondensate dissolution were strongly affected by the presence of salt. For both formation and dissolution, 10 µM NaCl was able to disrupt nanocondensation. This trend was observed from 0.5 to 4 µM protein (Figure 3B). pTau exhibited similar formation trends, wherein 10 µM salt was sufficient to disrupt nanocondensation (Figure 3C, top). However, nanocondensate dissolution required 1000× the concentration of salt (Figure 3C, bottom).

### 3.4. P301S Tau Exhibits Distinct Dissolution Properties Relative to WT and pTau

**Figure 4:**
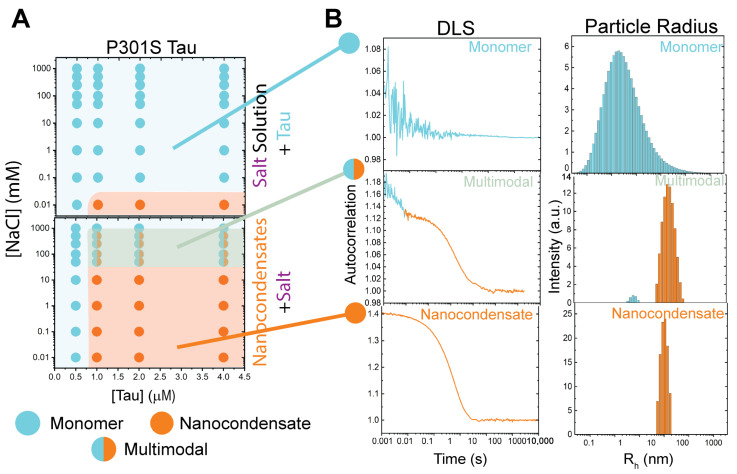
Formation and dissolution of P301S Tau nanocondensates, modulated by salt. (**A**) P301S condensate formation (top) and dissolution (bottom) in the presence of salt. (**B**) Autoattenuation and peak populations observed via DLS reveal monomer (blue), multimodal (blue to orange), and nanocondensate (orange) populations of P301S Tau.

P301S Tau was assessed similarly to WT and pTau. The formation of P301S nanocondensates followed the same trends as WT and pTau, but were only able to form condensates starting from 1 µM protein concentration (Figure 4A, top). The dissolution of P301S Tau resulted in a multimodal intermediate with the addition of 50–500 mM NaCl (Figure 4B, middle panel). P301S nanocondensate dissolution also required the highest concentration of salt of the three Tau variants (1 M NaCl).

## 4. Discussion

Condensation plays a considerable role in the function and dysfunction of Tau. The sequestration and concentration of Tau and its associated proteins into membrane-less compartments localize biochemical reactions [2,3,4,38], but the dysregulation of these condensates can lead to the formation of toxic specie linked to amyloidal aggregation [9,10,23,24,42,43,44]. Understanding the forces that drive and stabilize condensates will allow us to unravel differences between physiologic and pathologic condensates. In this study, we characterized WT, pTau, and P301S Tau condensate formation and dissolution.

Here, we demonstrated that all Tau variants can exist as monomers, nanocondensates, and microcondensates, or a combination of these states (Figure 1B). In our experimental conditions, WT Tau microcondensates were primarily stabilized by electrostatic interactions (Figure 2B). Our experiments used HEPES buffer at pH 8 because Tau LLPS is favored at alkaline pH [42,45]. Importantly, our buffer did not contain crowding agents, such as polyethylene glycol (PEG) or dextran. We chose these solution conditions to observe the electrostatic effects of salt on the protein itself, without confounding our observations with the physical (excluded volume) and chemical (attractive/repulsive) interactions between molecular crowders and the protein.

WT Tau microcondensates were dissolved by the addition of salt, reverting condensates to monomers. Interestingly, the DLS peak observed when measuring the dissolved Tau solution was broad (Figure 2B) compared to purely monomeric Tau (Figure 1B). The dissolved solution contained both HEPES buffer and salt, which may broaden the peak due to the decreased signal-to-noise ratio, as seen in Figure 4B. However, this does not completely exclude the possibility that the dissolution of condensates may revert the protein into dimeric or small oligomers that are not as easily distinguishable from monomers via DLS. We describe this population as ‘monomer’ due to the characteristics of the autocorrelation curve, but the exact protein state is not fully determined.

All three forms of Tau exhibited similar condensation formation properties in the presence of increased salt concentration (Figure 3B,C, and Figure 4).It is known that pTau microcondensate formation is altered compared to WT [3,21,22,23,42,45,46]; however, the formation of nanocondensates was relatively the same for both protein variants in solution conditions of the same ionic strength (Figure 3B,C, despite the change in charge distribution of pTau due to hyperphosphorylation (Figure 1A)). This suggests that, at the nanocondensate level, the forces driving Tau condensation were not significantly affected by changes in charge distribution caused by phosphorylation. Another noteworthy observation is that P301S Tau required a higher protein concentration for nanocondensation, indicating a decrease in condensation propensity. P301S has the same charge distribution as WT Tau, with only one missense mutation at residue 301. This mutation, however, may have significant consequences for intermolecular interactions. A mutation from proline to serine has significant secondary structural implications, as proline is known to disrupt helix and beta sheet structures [47,48,49]. Although Tau is an intrinsically disordered protein, condensation is a series of localized transient interactions [12,33,34,35,36,37] that involve the formation of temporary structures, a process that can be significantly affected by secondary structure propensities.

The dissolution of WT, pTau, and P301S condensates provides insights into the forces that stabilize condensation. WT Tau nanocondensates readily dissolves into monomeric species in the presence of low-concentration salt. pTau, however, requires 1000-fold salt concentration, compared to WT, to reverse nanocondensation. The significant increase in electrostatic screening requirement to reverse condensation may be caused by th altereded charge distribution due to phosphorylation. However, the most striking result came from P301S, which significantly resisted condensate reversion and formed multimodal intermediates. The concentration at which P301S fully reverted to monomeric form was 1 M salt: 100,000-fold salt concentration required as compared to WT. As mentioned above, P301S and WT Tau share charge distributions, and the only difference between the two forms is the single point mutation. The multimodal form of P301S exhibits two populations: one at around monomeric size, and the other at nanocondensate size. This suggests that P301S condensates resist complete dissolution by electrostatic screening, potentially due to the formation of alternate nanocondensate states exhibiting differential stabilities.

## 5. Conclusions

Our results demonstrate the subtle and complex balance in forces that govern the initiation, formation, and stabilization of protein condensates. Condensates that form from pathologic variants of Tau are more resistant to reversony, which alludes to differences in condensate properties that may define the boundaries between physiological and pathological condensates. Further research may uncover means by which the transition to disease states may be inhibited, leading to new therapeutic strategies that include the small molecule targeting of pathological condensates.

## Data Availability

Source data for plots, raw data for counts and intensity measurements, and uncropped gel images generated in this study are available from the corresponding authors upon request.

## Supplementary Materials

The following supporting information can be downloaded at: https://www.mdpi.com/article/10.3390/biom15030406/s1, Figure S1: Purified Tau variants; Figure S2: Mass spectrometry verification of hyperphosphorylation.
