# Supplementary material for: Electrostatic Effects on Tau Nanocondensates
Source: Biomolecules. 2025 Mar 12;15(3):406. doi: 10.3390/biom15030406 (PMC11940141; doi:10.3390/biom15030406)
Supplement: Supplementary file 1 [file biomolecules-15-00406-s001.zip › biomolecules-3488895-supplementary.pdf]

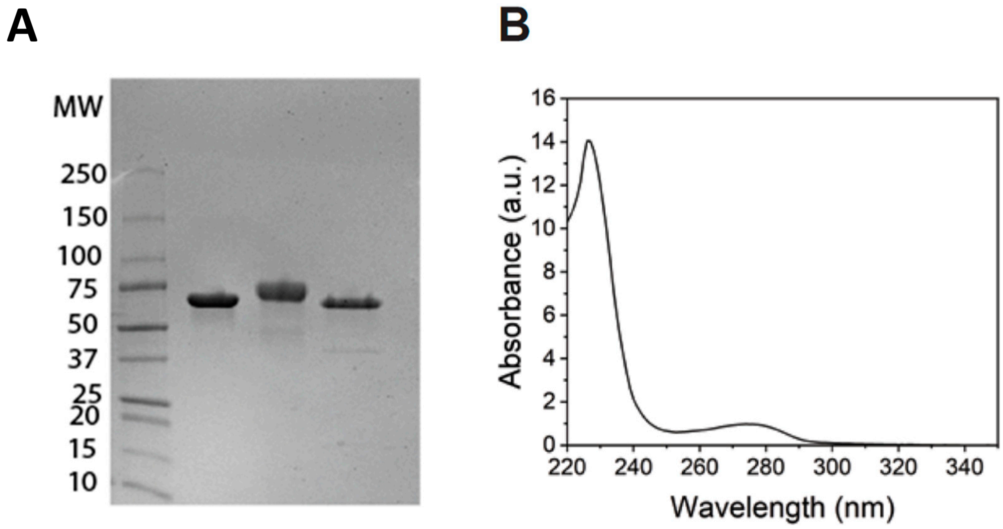

Supplementary Figure 1: Purified Tau Variants. **A**. SDS-PAGE of purified Tau variants. From left to right, 50 µM WT, pTau, and P301S Tau. **B**. UV Absorbance spectrum of 50µM purified WT Tau.

A

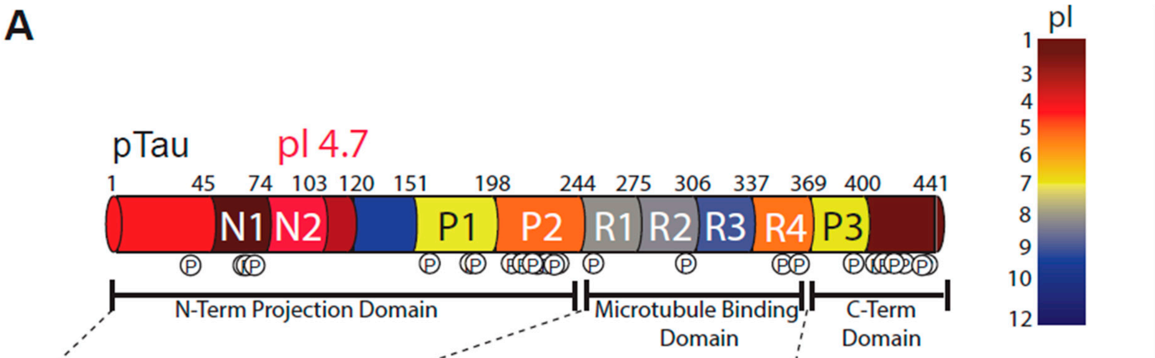

Peptide sequence	Phos. Site
TmHQDQEGDtDAGLKESPL	T39
DAGLKESPLQtPTE	T50
DAKStPTAEDVTAPLV	T69
STPtAEDVTAPLVDEGAPGK	T71
TKIAtPR	T153
IPAKtPPAPK	T175
TPPAPKtPPSSGEPPK	T181
SGYSSPGsPGtPGSR	S202+T205
SGYSSPGSPGtPGSR	T205
sRTPSLPtPPTREPK	S210+T217
SRtPSLPTPPTR	T212
SRtPsLPTPPTR	T212+S214
SRtPSLPtPPTREPK	T212+T217
VAVVRtPPKsPSSAK	T231+S235
TPPKsPSSAK	S235
LQtAPVPmPDLK	T245
DNIKHVPGGGsVQIVYKPV	S305
DRVQsKIGSL	S352
DRVQSKIGsL	S356
DHGAEIVYKsPVVSG	S396
TDHGAEIVYKsPVVSGDTsPR	S396+S404

Peptide sequence	Phos. Site
LQtAPVPmPDLK	T245
DNIKHVPGGGsVQIVYKPV	S305
DRVQsKIGSL	S352
DRVQSKIGsL	S356

Peptide sequence	Phos. Site
DHGAEIVYKsPVVSG	S396
TDHGAEIVYKsPVVSGDTsPR	S396+S404
DtSPRHLSNVSSTGSIDmV	T403
DtSPRHLSNVSSTGsIDmV	T403+S416
SPVVSGDTsPR	S404
DTsPRHLsNVSSTGSI	S404+S409
HLsNVSSTGSIDmVDSPQLATLADE\	S409
HLSNVsSTGSIDmVDSPQLATLADE\	S412
SNVSsTGSIDMVDSPQLATL	S413
DTSPRHLSNVSSTGsIDmV	S416
SNVSSTGsIDmVDsPQLATL	S416+S422
DsPQLATLA	S422
HLSNVSSTGSIDMVDSPQLAtLADE\	T427
DEVsASLAKQGL	S433

B

IPAKtPPAPK
Phos. Site T175

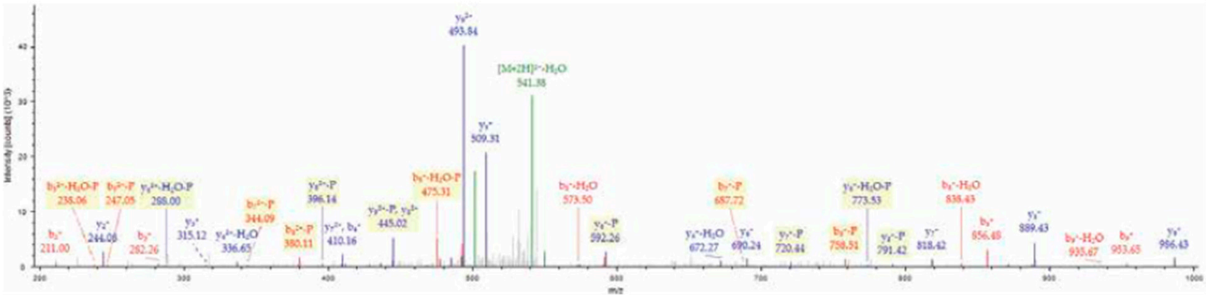

Supplementary Figure 2: Mass Spectrometry Verification of Hyperphosphorylation. **A**. Tau Domain organization of pTau, including a table of exact phosphorylation sites. **B**. MS/MS spectra of peptide containing phosphorylated T175.
