# Supplementary figures and images for: Electrostatic Effects on Tau Nanocondensates
Source: Biomolecules. 2025 Mar 12;15(3):406. doi: 10.3390/biom15030406 (PMC11940141; doi:10.3390/biom15030406)

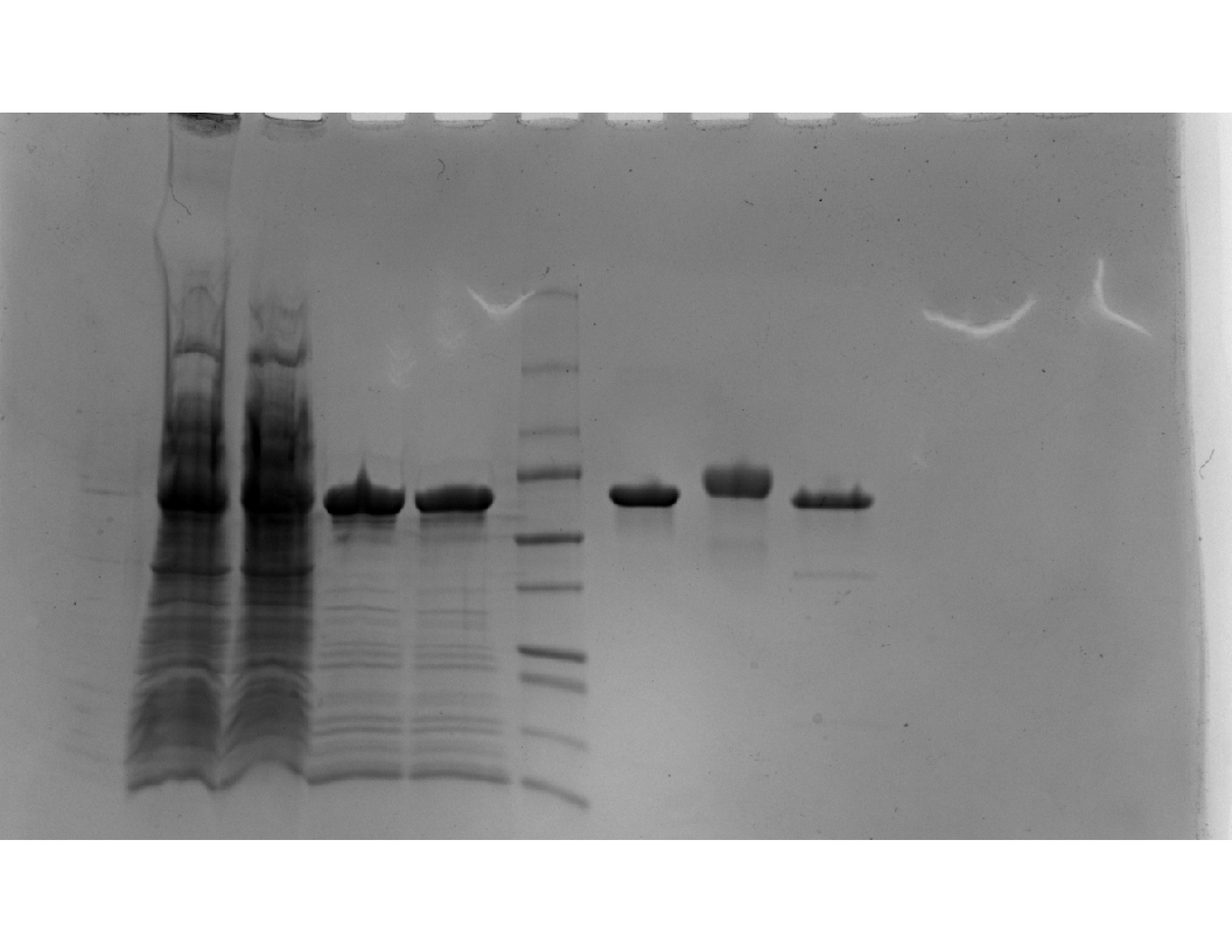

Supplement: Supplementary file 1 [file biomolecules-15-00406-s001.zip › biomolecules-3488895 - original image.pdf]
